# Optimal voluntary and mandatory insect repellent usage and emigration strategies to control the chikungunya outbreak on Reunion Island

**DOI:** 10.7717/peerj.10151

**Published:** 2020-12-17

**Authors:** Sylvia R.M. Klein, Alex O. Foster, David A. Feagins, Jonathan T. Rowell, Igor V. Erovenko

**Affiliations:** 1 Department of Mathematics, St. Mary’s College of Maryland, St. Mary’s City, MD, USA; 2 Department of Mathematics and Statistics, Coastal Carolina University, Conway, SC, USA; 3 Department of Mathematics, St. Mary’s University, San Antonio, TX, USA; 4 Department of Mathematics and Statistics, University of North Carolina at Greensboro, Greensboro, NC, USA

**Keywords:** Chikungunya, Epidemiology, Game theory, Herd immunity, Nash equilibrium, Reunion Island

## Abstract

In 2005, a chikungunya virus outbreak devastated the tropical island of Reunion, infecting a third of the total population. Motivated by the Reunion Island case study, we investigate the theoretic potential for two intervention measures under both voluntary and mandatory protocols to control a vector-borne disease when there is risk of the disease becoming endemic. The first measure uses insect repellent to prevent mosquito bites, while the second involves emigrating to the neighboring Mauritius Island to avoid infection. There is a threshold on the cost of using repellent above which both voluntary and mandatory regimes find it optimal to forgo usage. Below that threshold, mandatory usage protocols will eradicate the disease; however, voluntary adoption leaves the disease at a small endemic level. Emigrating from the island to avoid infection results in a tragedy-of-the-commons effect: while being potentially beneficial to specific susceptible individuals, the remaining islanders paradoxically face a higher risk of infection. Mandated relocation of susceptible individuals away from the epidemic is viable only if the cost of this relocation is several magnitudes lower than the cost of infection. Since this assumption is unlikely to hold for chikungunya, it is optimal to discourage such emigration for the benefit of the entire population. An underlying assumption about the conservation of human-vector encounter rates in mosquito biting behavior informs our conclusions and may warrant additional experimental verification.

## Introduction

Reunion Island is a tropical island located in the Indian Ocean 500 miles east of Madagascar and approximately 150 miles southwest of Mauritius. The island was devastated by a major chikungunya outbreak in 2005–2006, when approximately 266 thousand of the 785 thousand inhabitants were infected, causing over 200 deaths (Yakob & Clements, 2013). In the aftermath of that outbreak, the chikungunya virus spread from Africa to Europe, USA, and Australia, and although the incidence levels of this disease remain low, its potential to cause future outbreaks in these areas is cause for concern. In this paper, we investigate the viability of voluntary participation in personal protective measures (mosquito repellent and emigration) against diseases like chikungunya on Reunion Island by constructing a game-theoretic model in which individual strategic payoffs are compared against the average population payoff.

Chikungunya virus (CHIKV) is an *Alphavirus* in the Togaviridae family, similar to Dengue fever and Zika virus (Leao et al., 2018; Prow et al., 2018; Pile et al., 1999). It is a vector-borne virus spread through bites by the females of *Aedes aegypti* and *Aedes albopictus* mosquitoes. After a bite, there is a latency period for both humans and mosquitoes: it can take between 2 to 6 days for symptoms to develop and for an individual to become infectious (Fourie & Morrison, 1979). The major symptoms associated with CHIKV are fever, rash, arthritis, headache, and nausea (Fourie & Morrison, 1979). The defining characteristic of CHIKV is the persistence of arthritis for years after the initial infection (Leao et al., 2018). A small percentage of people infected with CHIKV, however, never develop symptoms of the disease (Darrigo, De Sant’Anna Carvalho & Machado, 2018). Humans are no longer infectious about a week and a half after the initial infection, but may still be symptomatic. Recovered individuals acquire lifelong immunity from future infections (Centers for Disease Control and Prevention, 2018b). There is no vaccine to prevent or medicine to treat chikungunya virus (Centers for Disease Control and Prevention, 2018b). The most effective way to prevent infection from CHIKV is to prevent mosquito bites, for example, by using insect repellent (Centers for Disease Control and Prevention, 2018a).

Chikungunya was first isolated in 1952–1953 in Tanzania (Robinson, 1955). The name translates to the native term for “that which bends up” (Seneviratne, Gurugama & Perera, 2007). There were limited outbreaks between the initial discovery of the disease and a worldwide outbreak that occurred in 2004–2005 (Darrigo, De Sant’Anna Carvalho & Machado, 2018). This outbreak started in Kenya and spread to the surrounding islands including Mauritius, Rodrigues, The Seychelles, Mayotte, Madagascar and Reunion Island (Renault et al., 2012). From these islands, it spread to other regions of the world—chikungunya virus is now present on every continent except Antarctica—most likely carried by tourists. The disease impacted Reunion Island most severely: a third of the population became infected, unusually severe forms were present, and the first occurrences of maternal-neonatal transmission were documented (Borgherini et al., 2008). This severity of impact may be attributed to an increase in travel between islands and the climate of the region at the time of the epidemic (Borgherini et al., 2008; Thiberville et al., 2013).

While the severity of the Reunion chikungunya outbreak may seem like an isolated event, vector-borne diseases such as malaria and dengue are becoming an increasingly prevalent public health issue in today’s society. In the United States there has been a 23-fold increase of vector-borne disease cases in the past ten years (Rosenberg et al., 2018). There are now 16 vector-borne diseases widely distributed in the United States, all of which are resistant to control, and only one of these (Yellow Fever) has an FDA-approved vaccine (Rosenberg et al., 2018). Even though there are limited cases of CHIKV in the United States and its territories, the disease is becoming more persistent: the number of national cases and distribution are increasing, and the range of the mosquitoes that transmit CHIKV has spread to 38 states as of 2016 (Rosenberg et al., 2018).

Due to the transmission patterns of mosquito-borne diseases and the lack of sufficient vector control to eradicate such diseases, individuals often have to rely on voluntary participation in personal protection measures. Unfortunately, individual self-interest in protection against an infectious disease does not necessarily correspond to the desired outcome for society (Galvani, Reluga & Chapman, 2007), namely eradication of the disease. The effect of potentially selfish human behavior on the spread of infectious diseases only recently begun to receive attention, forming a new field of *behavioral epidemiology*; see Manfredi & D’Onofrio (2013) for a review of behavioral epidemiology.

Originally designed for the field of economics (Von Neumann & Morgenstern, 1944), game theory has since been used to model many biological phenomena (Maynard Smith, 1982; Hofbauer & Sigmund, 1998; Cressman, 2003; Vincent & Brown, 2005; Broom & Rychtář, 2013), including individual-level vaccination decisions (Bauch & Earn, 2004). In a vaccination game, a selfish individual seeks to maximize its benefit, or rather to minimize the potential loss resulting from either employing a potentially costly protective measure or facing the consequences of the disease. As the likelihood of contracting the disease is dependent upon the behavior of others within the at-risk population, the resulting strategic interactions between individuals can be modeled using game theory. Game-theoretic frameworks have been adopted to studying optimal individual vaccination strategies for smallpox (Bauch, Galvani & Earn, 2003), influenza (Galvani, Reluga & Chapman, 2007; Shim et al., 2012a), rubella (Shim, Kochin & Galvani, 2009), measles (Shim et al., 2012b), toxoplasmosis (Sykes & Rychtář, 2015), Ebola (Brettin et al., 2018), cholera (Kobe et al., 2018), meningitis (A Martinez, J Machado, E Sanchez, I Erovenko, 2019, unpublished data), hepatitis B (Chouhan et al., 2020), monkeypox (Bankuru et al., 2020), poliomyelitis (Cheng et al., 2020), and typhoid fever (Acosta-Alonzo et al., 2020). It has also been applied to other personal protective measures such as insecticide-treated cattle (Crawford et al., 2015), mosquito repellent (Dorsett et al., 2016), insecticide-treated bed nets (Broom, Rychtář & Spears-Gill, 2016), clean water (Kobe et al., 2018), and clean injecting equipment (K Scheckelhoff, A Ejaz, I Erovenko, 2019, unpublished data). For an extensive review of behavior-linked vaccination models, see Wang et al. (2016).

In this paper, we investigate the potential effects of voluntary and government-mandated participation in utilizing the insect repellent as a protective measure against a disease such as chikungunya on Reunion Island. We also analyze the effect of emigration to a neighboring island (Mauritius) on the spread of chikungunya among the remaining population of Reunion Island. We find that the latter protocol has a paradoxically worsening of outcomes for the non-participating population potentially as a consequence of the non-responsiveness in the mosquitoes feeding behavior to decreases in the human population relative to other blood sources.

## Methods

We adopt a version of the epidemiological model of the chikungunya outbreak on Reunion Island by Yakob & Clements (2013) by adding population dynamics (birth and death demographic processes) for both humans and mosquitoes and strategically-linked parameters so that the disease potentially may establish itself endemically. This assumption then permits us to use the framework of Dorsett et al. (2016), Amaku et al. (2014), and Bauch & Earn (2004). All human inhabitants of the island (*N*) are divided into 5 compartments: individuals susceptible to chikungunya (*S*); exposed individuals (*E*), who had been bitten by an infected mosquito and acquired the disease; symptomatic infectious individuals (*I*), who developed the symptoms of the disease and became infectious to biting mosquitoes; asymptomatic infectious individuals (*I*_*a*_), who became infectious but did not develop symptoms; and recovered individuals (*R*), who recovered from chikungunya and acquired immunity. The mosquito population is divided into 3 compartments: susceptible mosquitoes (*X*); exposed mosquitoes (*Y*), who bit an infected human and were exposed to the pathogen; and infectious mosquitoes (*Z*), who may infect humans by biting susceptible individuals. We did not consider in this model the full life-cycle of mosquitoes, such as egg and larval stages, because we did not incorporate mosquito population control as one of the measures to fight chikungunya.

New individuals enter the susceptible part of the population at a rate Λ_1_ due to birth or immigration; there is a natural per capita human mortality *μ*_1_. Similarly, new mosquitoes are recruited into the susceptible compartment at a rate Λ_2_, and there is a natural per capita mosquito mortality *μ*_2_. We disregard the human disease-induced mortality because it is low, and doing so allows us to compute endemic equilibria analytically.

Susceptible humans who are bitten by infectious mosquitoes become exposed. The force of infection *f*_1_, which is the rate at which susceptible individuals move to the exposed class, depends on the density of susceptible humans *S*∕*N* (i.e., the probability that an infectious mosquito bites a susceptible individual), the number of infectious mosquitoes *Z*, and the mosquito-to-human transmission coefficient *β*_1_. We assume that mosquitoes have a consistent average number of encounters with humans over a given time span, and that repellent usage directly decreases the force of infection by deterring biting upon encounter. Similarly, susceptible mosquitoes who bite infectious humans become exposed. The force of infection *f*_2_, which is the rate at which susceptible mosquitoes move to the exposed compartment, depends on the the density of infectious humans (*I* + *I*_*a*_)∕*N* (i.e., the probability that a mosquito bites an infectious individual), the number of susceptible mosquitoes *X*, and the human-to-mosquito transmission coefficient *β*_2_.

Exposed humans become infectious (after a latency period) at a rate *λ*_1_; a proportion *ϕ* of infectious individuals develop symptoms of the disease. Exposed mosquitoes become infectious at a rate *λ*_2_.

Infectious humans (both symptomatic and asymptomatic) recover at a rate *γ* and acquire immunity from future infections. The lifespan of a mosquito is too short to recover; an infectious mosquito remains as such until it dies.

**Figure 1:**
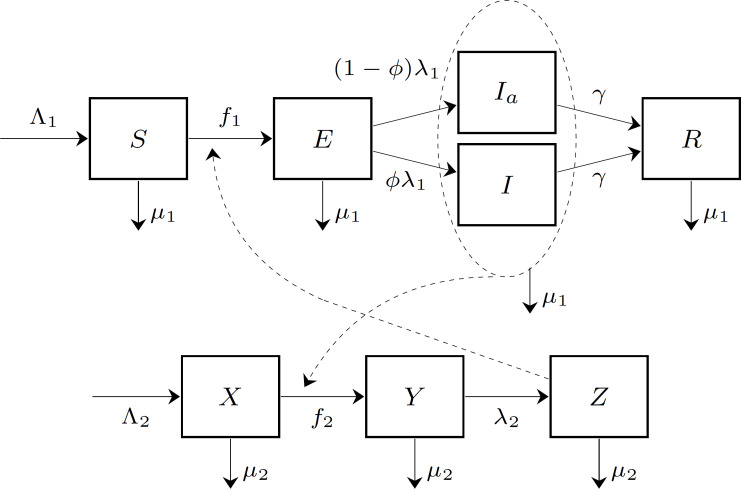
The compartment model of chikungunya virus transmission on Reunion Island. The human population is divided into five compartments: susceptible (*S*), exposed (*E*), symptomatic infectious (*I*), asymptomatic infectious (*I*_*a*_), and recovered (*R*). The mosquito population is divided into three compartments: susceptible (*X*), exposed (*Y*), and infectious (*Z*). The forces of infection on human and mosquito populations, *f*_1_ and *f*_2_, respectively, are population frequency-dependent functions of the state variables.

Figure 1 shows a diagram for the chikungunya transmission model on Reunion Island. The parameters of the epidemiological model are summarized in Table 1. The table includes the baseline value of the mosquito-to-human transmission parameter, denoted by ${\beta }_{1}^{0}$. Later this parameter will be affected by an intervention measure (insect repellent), and hence it will become a function of the level of insect repellent usage, given by (7).

**Table 1:** Summary of the model parameters.

Symbol	Meaning	Value	Source
${\beta }_{1}^{0}$	Mosquito-to-human transmission	0.37	Dumont, Chiroleu & Domerg (2008)
*β*_2_	Human-to-mosquito transmission	0.37	Dumont, Chiroleu & Domerg (2008)
*γ*	Human recovery rate	0.14	Yakob & Clements (2013)
Λ_1_	Human birth rate	3.58	Assumed
Λ_2_	Mosquito birth rate	4.76 × 10^3^	Assumed
*λ*_1_	Rate of humans becoming infectious	0.5	Yakob & Clements (2013)
*λ*_2_	Rate of mosquitoes becoming infectious	0.5	Yakob & Clements (2013)
*μ*_1_	Human natural death rate	3.58 × 10^−5^	Assumed
*μ*_2_	Mosquito natural death rate	0.05	Yakob & Clements (2013)
*ϕ*	Proportion of hosts that develop symptoms	0.97	Yakob & Clements (2013)

The dynamics of the compartment model in Fig. 1 is described by the following system of differential equations:

(1) \begin{eqnarray*} \frac{\mathrm{d}S}{\mathrm{d}t} ={\Lambda }_{1}- \frac{{\beta }_{1}SZ}{N} -{\mu }_{1}S, \frac{\mathrm{d}E}{\mathrm{d}t} = \frac{{\beta }_{1}SZ}{N} -{\lambda }_{1}E-{\mu }_{1}E, \frac{\mathrm{d}I}{\mathrm{d}t} =\phi {\lambda }_{1}E-\gamma I-{\mu }_{1}I, \frac{\mathrm{d}{I}_{a}}{\mathrm{d}t} =(1-\phi ){\lambda }_{1}E-\gamma {I}_{a}-{\mu }_{1}{I}_{a}, \frac{\mathrm{d}R}{\mathrm{d}t} =\gamma (I+{I}_{a})-{\mu }_{1}R, \frac{\mathrm{d}X}{\mathrm{d}t} ={\Lambda }_{2}- \frac{{\beta }_{2}X(I+{I}_{a})}{N} -{\mu }_{2}X, \frac{\mathrm{d}Y}{\mathrm{d}t} = \frac{{\beta }_{2}X(I+{I}_{a})}{N} -{\lambda }_{2}Y-{\mu }_{2}Y,\text{and} \frac{\mathrm{d}Z}{\mathrm{d}t} ={\lambda }_{2}Y-{\mu }_{2}Z.\end{eqnarray*}

The disease-free equilibrium (DFE) of this system is given by

(2) \begin{eqnarray*} \left( {S}^{0},{E}^{0},{I}^{0},{I}_{a}^{0},{R}^{0},{X}^{0},{Y}^{0},{Z}^{0} \right) = \left( \frac{{\Lambda }_{1}}{{\mu }_{1}} ,0,0,0,0, \frac{{\Lambda }_{2}}{{\mu }_{2}} ,0,0 \right) .\end{eqnarray*}

To compute the basic reproduction number *R*_0_, we use the next-generation matrix approach (Van den Driessche & Watmough, 2002). To simplify this computation, we temporarily combined the symptomatic and asymptomatic infectious compartments into one infectious compartment *I* + *I*_*a*_: individuals in both *I* and *I*_*a*_ compartments have identical contributions to the dynamics of chikungunya. We order the compartments that contribute to new infections as follows: *E*, *I* + *I*_*a*_, *Y*, and *Z*. Then the vector of the rates of appearance of new infections in these four compartments $\mathcal{F}$ and the vector of the rates of transfer of existing infections between these four compartments $\mathcal{V }$ are given by

(3) \begin{eqnarray*}\mathcal{F}= \left[ \begin{array}{@{}c@{}} \displaystyle \frac{{\beta }_{1}SZ}{N} \\ \displaystyle 0\\ \displaystyle \frac{{\beta }_{2}X(I+{I}_{a})}{N} \\ \displaystyle 0 \end{array} \right] \text{and} \mathcal{V }= \left[ \begin{array}{@{}c@{}} \displaystyle {\lambda }_{1}E+{\mu }_{1}E\\ \displaystyle -{\lambda }_{1}E+\gamma (I+{I}_{a})+{\mu }_{1}(I+{I}_{a})\\ \displaystyle {\lambda }_{2}Y+{\mu }_{2}Y\\ \displaystyle -{\lambda }_{2}Y+{\mu }_{2}Z \end{array} \right] .\end{eqnarray*}

The matrices *F* and *V* are the Jacobians of $\mathcal{F}$ and $\mathcal{V }$ respectively, evaluated at DFE; they are given by

(4) \begin{eqnarray*}F= \left[ \begin{array}{@{}cccc@{}} \displaystyle 0&\displaystyle 0&\displaystyle 0&\displaystyle {\beta }_{1}\\ \displaystyle 0&\displaystyle 0&\displaystyle 0&\displaystyle 0\\ \displaystyle 0&\displaystyle \frac{{\Lambda }_{2}{\beta }_{2}{\mu }_{1}}{{\Lambda }_{1}{\mu }_{2}} &\displaystyle 0&\displaystyle 0\\ \displaystyle 0&\displaystyle 0&\displaystyle 0&\displaystyle 0 \end{array} \right] \text{and} V= \left[ \begin{array}{@{}cccc@{}} \displaystyle {\lambda }_{1}+{\mu }_{1}&\displaystyle 0&\displaystyle 0&\displaystyle 0\\ \displaystyle -{\lambda }_{1}&\displaystyle \gamma +{\mu }_{1}&\displaystyle 0&\displaystyle 0\\ \displaystyle 0&\displaystyle 0&\displaystyle {\lambda }_{2}+{\mu }_{2}&\displaystyle 0\\ \displaystyle 0&\displaystyle 0&\displaystyle -{\lambda }_{2}&\displaystyle {\mu }_{2} \end{array} \right] .\end{eqnarray*}

The basic reproduction number is the spectral radius of the matrix *FV*^−1^; it is given by

(5) \begin{eqnarray*}{R}_{0}= \frac{1}{{\mu }_{2}} \sqrt{ \frac{{\Lambda }_{2}{\beta }_{1}{\beta }_{2}{\mu }_{1}{\lambda }_{1}{\lambda }_{2}}{{\Lambda }_{1}(\gamma +{\mu }_{1})({\lambda }_{1}+{\mu }_{1})({\lambda }_{2}+{\mu }_{2})} }.\end{eqnarray*}

If *R*_0_ > 1, then the system converges to the endemic equilibrium (EE) given by

(6) \begin{eqnarray*}{S}^{\ast }= \frac{{\Lambda }_{1}-({\lambda }_{1}+{\mu }_{1}){E}^{\ast }}{{\mu }_{1}} , & {E}^{\ast }= \frac{{\Lambda }_{1}{\Lambda }_{2}{\beta }_{1}{\beta }_{2}{\mu }_{1}{\lambda }_{1}{\lambda }_{2}-{\Lambda }_{1}^{2}{\mu }_{2}^{2}({\lambda }_{1}+{\mu }_{1})({\lambda }_{2}+{\mu }_{2})(\gamma +{\mu }_{1})}{{\Lambda }_{2}{\beta }_{1}{\beta }_{2}{\mu }_{1}{\lambda }_{1}{\lambda }_{2}({\lambda }_{1}+{\mu }_{1})+{\Lambda }_{1}{\beta }_{2}{\mu }_{1}{\mu }_{2}{\lambda }_{1}({\lambda }_{1}+{\mu }_{1})({\lambda }_{2}+{\mu }_{2})} ,{I}^{\ast }= \frac{\phi {\lambda }_{1}{E}^{\ast }}{\gamma +{\mu }_{1}} ,{I}_{a}^{\ast }= \frac{(1-\phi ){\lambda }_{1}{E}^{\ast }}{\gamma +{\mu }_{1}} ,{R}^{\ast }= \frac{\gamma {\lambda }_{1}{E}^{\ast }}{{\mu }_{1}(\gamma +{\mu }_{1})} ,{X}^{\ast }= \frac{{\Lambda }_{1}{\Lambda }_{2}(\gamma +{\mu }_{1})}{{\beta }_{2}{\mu }_{1}{\lambda }_{1}{E}^{\ast }+{\Lambda }_{1}{\mu }_{2}(\gamma +{\mu }_{1})} ,{Y}^{\ast }= \frac{{\Lambda }_{2}{\beta }_{2}{\mu }_{1}{\lambda }_{1}{E}^{\ast }}{({\lambda }_{2}+{\mu }_{2})({\beta }_{2}{\mu }_{1}{\lambda }_{1}{E}^{\ast }+{\Lambda }_{1}{\mu }_{2}(\gamma +{\mu }_{1}))} ,\text{and}{Z}^{\ast }= \frac{{\Lambda }_{2}{\beta }_{2}{\mu }_{1}{\lambda }_{1}{\lambda }_{2}{E}^{\ast }}{{\mu }_{2}({\lambda }_{2}+{\mu }_{2})({\beta }_{2}{\mu }_{1}{\lambda }_{1}{E}^{\ast }+{\Lambda }_{1}{\mu }_{2}(\gamma +{\mu }_{1}))} \text{.}\end{eqnarray*}

In the game-theoretic models constructed in the next section, we will be assuming that the system has reached an endemic equilibrium. In particular, we will use the values from (6) for relevant compartment sizes.

## Results

We consider two intervention measures to fight the chikungunya outbreak on Reunion Island: (1) using insect repellent to prevent mosquito bites; and (2) emigrating to the neighboring Mauritius Island.

### Optimal levels of voluntary insect repellent usage

We adopt a modeling approach of Bauch & Earn (2004) and Dorsett et al. (2016). The strategy of an individual is the proportion of the day *r* ∈ [0, 1] the individual is protected from mosquito bites; the protection is granted by insect repellent. We assume that the repellent provides complete protection from mosquito bites while it is active. Since mosquitoes cannot bites humans while they are protected by the insect repellent, the mosquito-to-human transmission coefficient *β*_1_ becomes a function of *r*. If no protection is used (*r* = 0), then *β*_1_(0) is at its base value ${\beta }_{1}^{0}$ (given in Table 1). If humans are protected at all times (*r* = 1), then mosquitoes cannot bite these humans at all, and hence they cannot infect humans: *β*_1_(1) = 0. We therefore assume that the mosquito-to-human transmission coefficient is a linear function of *r* given by

(7) \begin{eqnarray*}{\beta }_{1}={\beta }_{1}^{0}(1-r).\end{eqnarray*}

**Figure 2:**
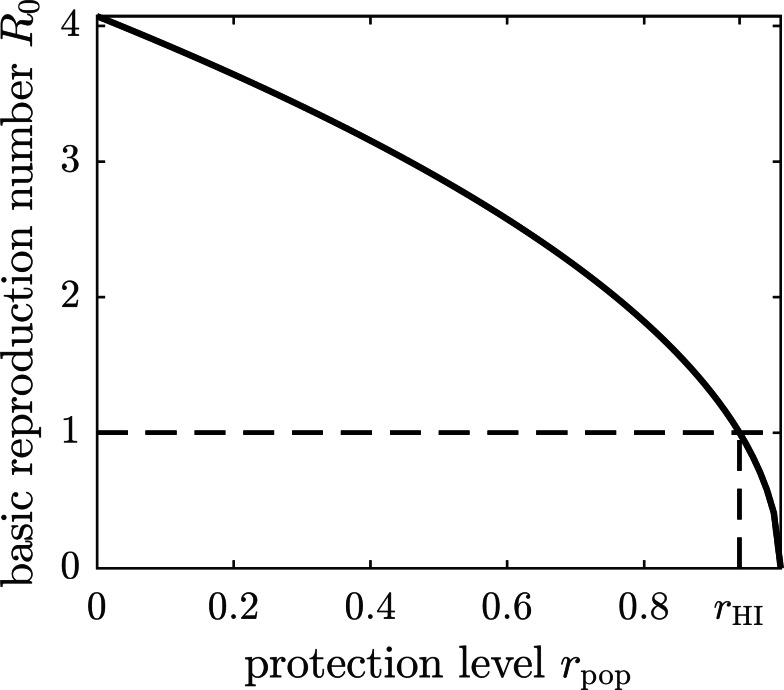
The graph of the basic reproduction number as a function of the population protection level *r*_pop_. The basic reproduction number is at its maximum value—given by (5)—when no insect repellent is used by susceptible individuals (*r* = 0), and it becomes zero if the population employs complete protection from mosquito bites (*r* = 1). The threshold for disease eradication (*R*_0_ = 1) is achieved at the herd immunity protection level *r*_HI_.

If all susceptible humans in the population adopt the same strategy *r*_pop_, then the basic reproduction number becomes a function of *r*_pop_ by substituting the expression (7) for *β*_1_ into (5). The graph of the basic reproduction number as a function of the population strategy *r*_pop_ is shown in Fig. 2. The herd immunity protection level *r*_HI_ is the population protection level that reaches the threshold *R*_0_ = 1 for disease eradication.

We define the utility function (expected payoff) of a susceptible individual using strategy *r* in a population that adopted strategy *r*_pop_ as

(8) \begin{eqnarray*}E(r,{r}_{\text{pop}})=-\pi (r,{r}_{\text{pop}}){C}_{i}-r{C}_{p},\end{eqnarray*}

where *C*_*i*_ is the cost of infection, *C*_*p*_ is the cost of complete protection through insect repellent, and *π*(*r*, *r*_pop_) is the probability of infection. The latter depends on the individual’s strategy *r* because it determines how often mosquitoes may bite the individual, and on the population strategy *r*_pop_ because it affects the prevalence of the disease (e.g., the number of infected mosquitoes). The outcome of a game does not change if the utility function is scaled, so we divide the right-hand side of (8) by *C*_*i*_ to obtain

(9) \begin{eqnarray*}E(r,{r}_{\text{pop}})=-\pi (r,{r}_{\text{pop}})-rC,\end{eqnarray*}

where *C* = *C*_*p*_∕*C*_*i*_ is the cost of complete protection relative to the cost of infection.

We next compute the probability of getting infected and becoming symptomatic as the transition probability from the susceptible compartment *S* to the symptomatic infectious compartment *I*. This probability is the product of the probability that a susceptible individual becomes exposed *f*_1_∕(*μ*_1_ + *f*_1_) multiplied by the probability that an exposed individual becomes symptomatically infected *ϕλ*_1_∕(*μ*_1_ + *ϕλ*_1_ + (1 − *ϕ*)*λ*_1_) = *ϕλ*_1_∕(*μ*_1_ + *λ*_1_):

(10) \begin{eqnarray*}\pi (r,{r}_{\text{pop}})= \left( \frac{{f}_{1}(r,{r}_{\text{pop}})}{{\mu }_{1}+{f}_{1}(r,{r}_{\text{pop}})} \right) \left( \frac{\phi {\lambda }_{1}}{{\mu }_{1}+{\lambda }_{1}} \right) ,\end{eqnarray*}

where

(11) \begin{eqnarray*}{f}_{1}(r,{r}_{\text{pop}})={\beta }_{1}^{0}(1-r) \frac{{Z}^{\ast }}{{N}^{\ast }} \end{eqnarray*}

is the force of infection, which depends on the individual protection level *r* and on the population protection level *r*_pop_. The individual protection level *r* determines the rate at which mosquitoes bite the individual ${\beta }_{1}^{0}(1-r)$. The population protection level *r*_pop_ affects the prevalence of the disease in the population; it determines the size of the compartment *Z*^∗^ via the substitution of the expression ${\beta }_{1}^{0}(1-{r}_{\text{pop}})$ for *β*_1_ into (6). In particular, *Z*^∗^ and *N*^∗^ do not depend on the individual protection level *r*.

To find the Nash equilibrium population protection level, we attempt to maximize the utility function (9) of a focal individual. Observe that

(12) \begin{eqnarray*}{f}_{1}(r,{r}_{\text{pop}})=(1-r){f}_{1}(0,{r}_{\text{pop}}),\end{eqnarray*}

and hence

(13) \begin{eqnarray*} \frac{\partial }{\partial r} {f}_{1}(r,{r}_{\text{pop}})=-{f}_{1}(0,{r}_{\text{pop}}).\end{eqnarray*}

It follows that

(14) \begin{eqnarray*} \frac{{\partial }^{2}}{\partial {r}^{2}} E(r,{r}_{\text{pop}})= \frac{2{\mu }_{1}{f}_{1}(0,{r}_{\text{pop}})^{2}}{({\mu }_{1}+{f}_{1}(r,{r}_{\text{pop}}))^{3}} \gt 0.\end{eqnarray*}

Consequently, the utility function is a convex function of *r*, and thus it attains its maximum value at one of the endpoints: *r* = 0 or *r* = 1.

This conclusion can be interpreted as follows. If the population repellent usage is sufficiently high, then the probability of getting infected is very low. A focal individual would rather bypass the potentially costly preventive measure and face the low morbidity risk instead. Hence individuals may improve their payoff by deviating from the population strategy (they should stop using repellent). On the other hand, if the population repellent usage is low, then the probability of getting infected is too high, and a focal individual should prefer to pay the cost of complete protection rather than face the high morbidity risk. In this case, individuals may also improve their payoff by deviating from the population strategy (they should use repellent 100% of the time).

So, if the population repellent usage is too high, then individuals would do better if they stop using repellent, and hence the population repellent usage will decrease. Conversely, if the population repellent usage is too low, then individuals would do better if they start using repellent 100% of the time, and hence the population repellent usage will increase. If the population repellent usage is “just right” (Nash equilibrium), then individuals cannot improve their payoffs by using repellent either less frequently or more frequently. We note that there is a presumption of the population-wide adoption of treatment rates in our model that is common to ESS-modeling; however, in situations such as (14), there is a potential implication that the population should in fact separate into distinct groups with different adoption rates. As it goes beyond the framework discussed here, we leave that investigation for future research.

The Nash equilibrium protection level of the population *r*_NE_ is thus a solution to the equation

(15) \begin{eqnarray*}E(0,{r}_{\text{NE}})=E(1,{r}_{\text{NE}})\end{eqnarray*}

or

(16) \begin{eqnarray*} \frac{{f}_{1}(0,{r}_{\text{NE}})}{{\mu }_{1}+{f}_{1}(0,{r}_{\text{NE}})} \frac{\phi {\lambda }_{1}}{{\mu }_{1}+{\lambda }_{1}} =C.\end{eqnarray*}

**Figure 3:**
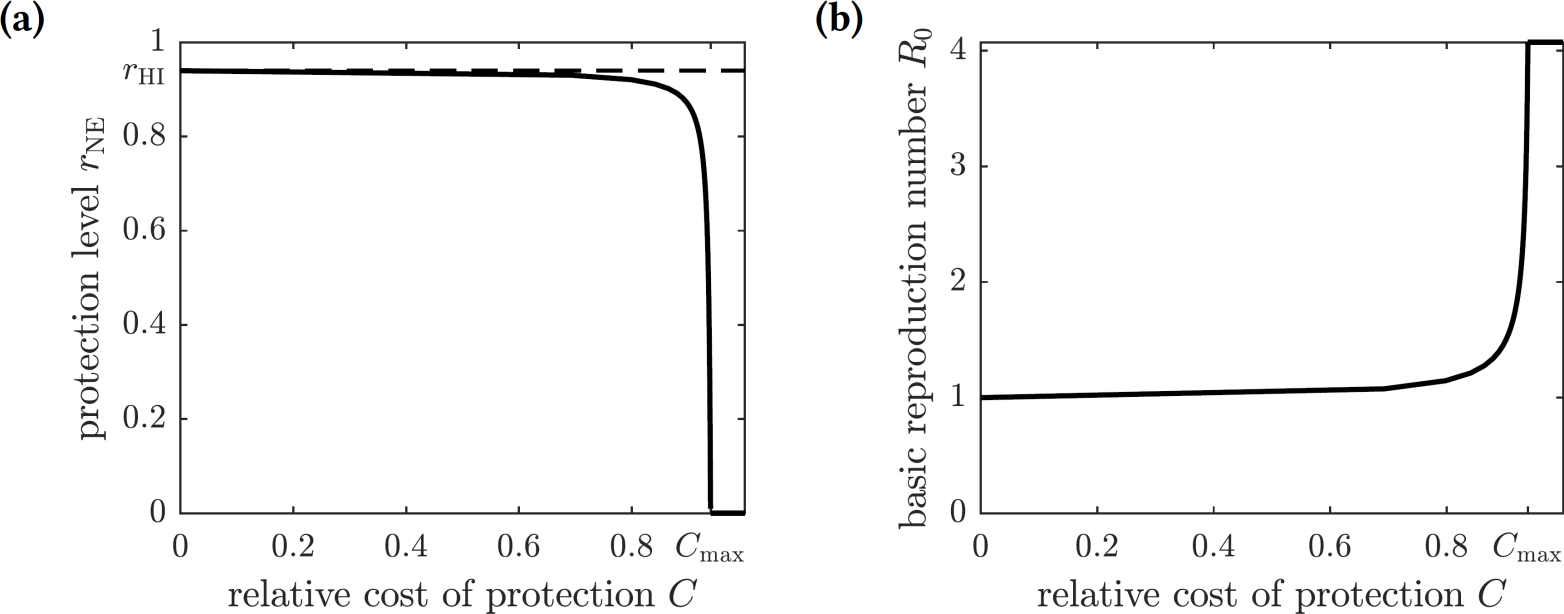
(A) The graph of the optimal level of population repellent usage *r*_NE_ as a function of the relative cost of protection. The optimal repellent usage reaches the herd immunity level only when *C* = 0. Everyone stops using repellent if its relative cost is too high: larger than the threshold value *C*_max_. (B) The graph of the basic reproduction number computed at the optimal population repellent usage level *r*_NE_ as a function of the relative cost of protection. When *C* = 0, the optimal protection level is equal to the herd immunity threshold, so *R*_0_ = 1. When the relative cost of protection exceeds the threshold value *C*_max_, the population stops using repellent, and the basic reproduction number reaches its maximum value.

The graph of the optimal (Nash equilibrium) repellent usage as a function of the relative cost of protection *C* is shown in Fig. 3A. The optimal repellent usage *r*_NE_ reaches the herd immunity *r*_HI_ level only when the cost of the protective measure relative to the cost of chikungunya infection is negligible (i.e., zero mathematically). The optimal repellent usage remains very close to the herd immunity level for a range of values of the relative cost *C*, and then drops off sharply. Once the relative cost of protection becomes too large (*C*_max_), then everyone stops using insect repellent because its high cost forces individuals to prefer to risk the cost of infection.

### Optimal levels of mandatory insect repellent usage

We now consider a scenario where an organization (e.g., the government) enforces the use of insect repellent in the population to fight chikungunya. The organization must balance the cost of prevention of the disease in the population and the cost of treatment of symptomatically infected individuals. On the one hand, every individual who utilizes insect repellent 100% of the time results in a cost *C*_*p*_ (same as the cost of voluntary complete protection). On the other hand, every symptomatically infected individual results in a cost *C*_*i*_.

The goal of the mandating organization is to find the repellent usage level for the population *r*_pop_ ∈ [0, 1] so that the expected payoff (negative of the total cost)

(17) \begin{eqnarray*}E({r}_{\text{pop}})=-{C}_{i}{I}^{\ast }-{r}_{\text{pop}}{C}_{p}{S}^{\ast }\end{eqnarray*}

is maximal. (Note that the equilibrium values *I*^∗^ and *S*^∗^ depend on *r*_pop_.) Here we analyze the case where the mandating organization addresses mosquito-to-human transmission by advising susceptible individuals to spray themselves with insect repellent. One may also consider an alternative scenario where the infectious individuals are using insect repellent to reduce the human-to-mosquito transmission.

As before, we scale the payoff function and consider

**Figure 4:**
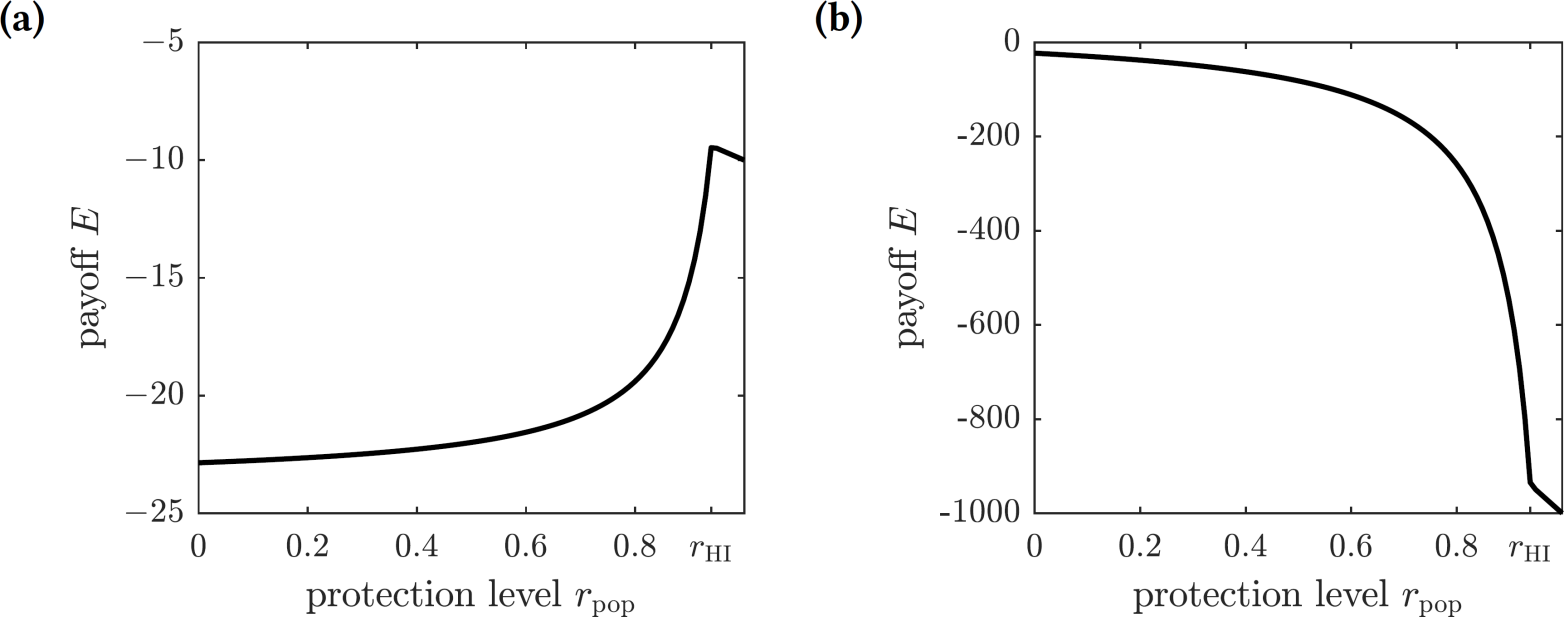
The graphs of the expected payoff function *E* given by the Eq. (18). (A) *C* = 0.0001, (B) *C* = 0.01. The graphs show two qualitatively different outcomes. If *C* < 0.00024 then mandating repellent usage necessary to reach the herd immunity threshold is most effective. If *C* > 0.00024 then no insect repellent should be used, and all efforts should be devoted to treating infected individuals.

(18) \begin{eqnarray*}E({r}_{\text{pop}})=-{I}^{\ast }-{r}_{\text{pop}}C{S}^{\ast },\end{eqnarray*}

where *C* = *C*_*p*_∕*C*_*i*_ is the relative cost of protection. The graphs of this function for different values of *C* are shown in Fig. 4. There are two possible outcomes: (1) the susceptible individuals should adopt the repellent usage level equal to that of the herd immunity threshold *r*_HI_, leading to the eradication of the disease; or (2) no insect repellent should be used, and it is more cost-effective to treat symptomatically infected individuals only. The first outcome occurs for sufficiently low values of *C* (less than 0.00024), and the second outcome occurs for greater values of *C* (greater than 0.00024); the threshold value of *C* separating the two outcomes was found numerically.

### Optimal levels of voluntary emigration

We are going to operate under the assumption that the chikungunya outbreak on Reunion Island did not affect the neighboring island of Mauritius Island, located 140 miles to the north-east of Reunion. Susceptible individuals—and only susceptible individuals, perhaps identified through a screening or quarantine procedure—may elect to emigrate from Reunion to Mauritius to protect themselves from the outbreak. To model the emigration as a potential personal protection measure against chikungunya, we allow residents of Reunion Island to leave the susceptible compartment of the epidemiological model at an emigration rate *ω*. This modification to (1) replaces the first equation describing the change in the susceptible population with

(19) \begin{eqnarray*} \frac{\mathrm{d}S}{\mathrm{d}t} ={\Lambda }_{1}- \frac{{\beta }_{1}SZ}{N} -{\mu }_{1}S-\omega S.\end{eqnarray*}

The DFE of the modified system is given by

(20) \begin{eqnarray*}({S}^{0},{E}^{0},{I}^{0},{I}_{a}^{0},{R}^{0},{X}^{0},{Y}^{0},{Z}^{0})= \left( \frac{{\Lambda }_{1}}{{\mu }_{1}+\omega } ,0,0,0,0, \frac{{\Lambda }_{2}}{{\mu }_{2}} ,0,0 \right) ,\end{eqnarray*}

and the corresponding basic reproduction number of the disease is

(21) \begin{eqnarray*}{R}_{0}= \frac{1}{{\mu }_{2}} \sqrt{ \frac{{\Lambda }_{2}{\beta }_{1}{\beta }_{2}{\lambda }_{1}{\lambda }_{2}({\mu }_{1}+\omega )}{{\Lambda }_{1}(\gamma +{\mu }_{1})({\lambda }_{1}+{\mu }_{1})({\lambda }_{2}+{\mu }_{2})} }.\end{eqnarray*}

**Figure 5:**
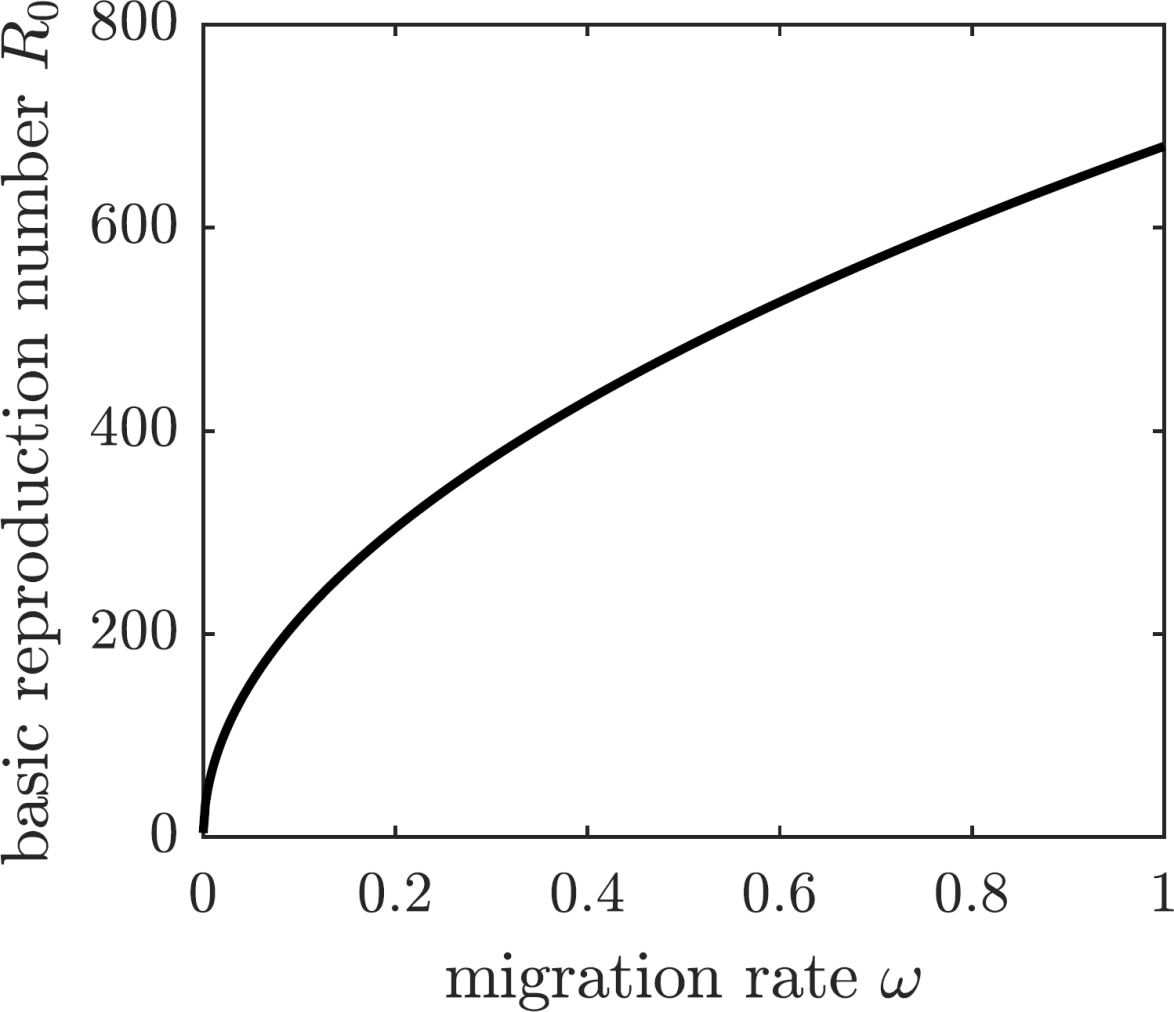
The graph of *R*_0_ as a function of *ω*. If susceptible individuals emigrate from Reunion Island, then the remaining inhabitants face an increased spread of the disease.

The graph of the basic reproduction number as a function of the emigration rate *ω* is shown in Fig. 5. It is an increasing function of *ω*, and hence emigrating susceptible individuals paradoxically make it worse for the remaining susceptible population. When the fresh blood supply is reduced due to emigration, susceptible mosquitoes are more likely to prey upon infectious humans, increasing the disease prevalence in the vector population. Consequently, the remaining susceptible human population is at an increased risk of contracting the disease from a mosquito bite. It follows that, while being potentially beneficial to specific individuals, voluntary emigration may result in a tragedy-of-the-commons effect for the remaining islanders.

To further investigate the effect of voluntary emigration on the chikungunya epidemic and whether (selfish) susceptible individuals should emigrate, we compute the EE values of all compartments in the model with emigration:

(22) \begin{eqnarray*}{N}^{\ast }= \frac{{\Lambda }_{1}{\mu }_{1}+\omega ({\lambda }_{1}+{\mu }_{1}){E}^{\ast }}{{\mu }_{1}({\mu }_{1}+\omega )} ,{S}^{\ast }= \frac{{\Lambda }_{1}-({\lambda }_{1}+{\mu }_{1}){E}^{\ast }}{{\mu }_{1}+\omega } ,{I}^{\ast }= \frac{\phi {\lambda }_{1}{E}^{\ast }}{\gamma +{\mu }_{1}} ,{I}_{a}^{\ast }= \frac{(1-\phi ){\lambda }_{1}{E}^{\ast }}{\gamma +{\mu }_{1}} ,{R}^{\ast }= \frac{\gamma {\lambda }_{1}{E}^{\ast }}{{\mu }_{1}(\gamma +{\mu }_{1})} ,{X}^{\ast }= \frac{{\Lambda }_{2}(\gamma +{\mu }_{1})({\Lambda }_{1}{\mu }_{1}+\omega ({\lambda }_{1}+{\mu }_{1}){E}^{\ast })}{d+{\mu }_{2}(\gamma +{\mu }_{1})({\Lambda }_{1}{\mu }_{1}+\omega ({\lambda }_{1}+{\mu }_{1}){E}^{\ast })} ,{Y}^{\ast }= \frac{{\Lambda }_{2}{\beta }_{2}{\mu }_{1}{\lambda }_{1}({\mu }_{1}+\omega ){E}^{\ast }}{({\lambda }_{2}+{\mu }_{2})[d+{\Lambda }_{1}{\mu }_{1}{\mu }_{2}(\gamma +{\mu }_{1})+{\mu }_{2}\omega ({\lambda }_{1}+{\mu }_{1})(\gamma +{\mu }_{1}){E}^{\ast }]} ,\text{and}{Z}^{\ast }= \frac{{\Lambda }_{2}{\beta }_{2}{\mu }_{1}{\lambda }_{1}{\lambda }_{2}({\mu }_{1}+\omega ){E}^{\ast }}{{\mu }_{2}({\lambda }_{2}+{\mu }_{2})[d+{\Lambda }_{1}{\mu }_{1}{\mu }_{2}(\gamma +{\mu }_{1})+{\mu }_{2}\omega ({\lambda }_{1}+{\mu }_{1})(\gamma +{\mu }_{1}){E}^{\ast }]} ,\end{eqnarray*}

where

(23) \begin{eqnarray*}d={\beta }_{2}{\lambda }_{1}{\mu }_{1}({\mu }_{1}+\omega ){E}^{\ast },\end{eqnarray*}

and *E*^∗^ is the solution to the quadratic equation

(24) \begin{eqnarray*}a{E}^{2}+bE+c=0\end{eqnarray*}

with coefficients

(25) \begin{eqnarray*}a=-{\mu }_{2}\omega ({\lambda }_{1}+{\mu }_{1})^{2}({\lambda }_{2}+{\mu }_{2})[{\beta }_{2}{\mu }_{1}{\lambda }_{1}({\mu }_{1}+\omega )+{\mu }_{2}\omega ({\lambda }_{1}+{\mu }_{1})(\gamma +{\mu }_{1})],b=-{\Lambda }_{2}{\beta }_{1}{\beta }_{2}{\mu }_{1}^{2}{\lambda }_{1}{\lambda }_{2}({\lambda }_{1}+{\mu }_{1})({\mu }_{1}+\omega )-2{\Lambda }_{1}{\mu }_{1}{\mu }_{2}^{2}\omega ({\lambda }_{1}+{\mu }_{1})^{2}({\lambda }_{2}+{\mu }_{2})(\gamma +{\mu }_{1}) -{\Lambda }_{1}{\beta }_{2}{\mu }_{1}^{2}{\mu }_{2}{\lambda }_{1}({\lambda }_{1}+{\mu }_{1})({\lambda }_{2}+{\mu }_{2})({\mu }_{1}+\omega ),\text{and}c={\Lambda }_{1}{\Lambda }_{2}{\beta }_{1}{\beta }_{2}{\mu }_{1}^{2}{\lambda }_{1}{\lambda }_{2}({\mu }_{2}+\omega )-{\Lambda }_{1}^{2}{\mu }_{1}^{2}{\mu }_{2}^{2}({\lambda }_{1}+{\mu }_{1})({\lambda }_{2}+{\mu }_{2})(\gamma +{\mu }_{1}).\end{eqnarray*}

The biologically meaningful root of this equation is given by

(26) \begin{eqnarray*}{E}^{\ast }= \frac{-b-\sqrt{{b}^{2}-4ac}}{2a} .\end{eqnarray*}

**Figure 6:**
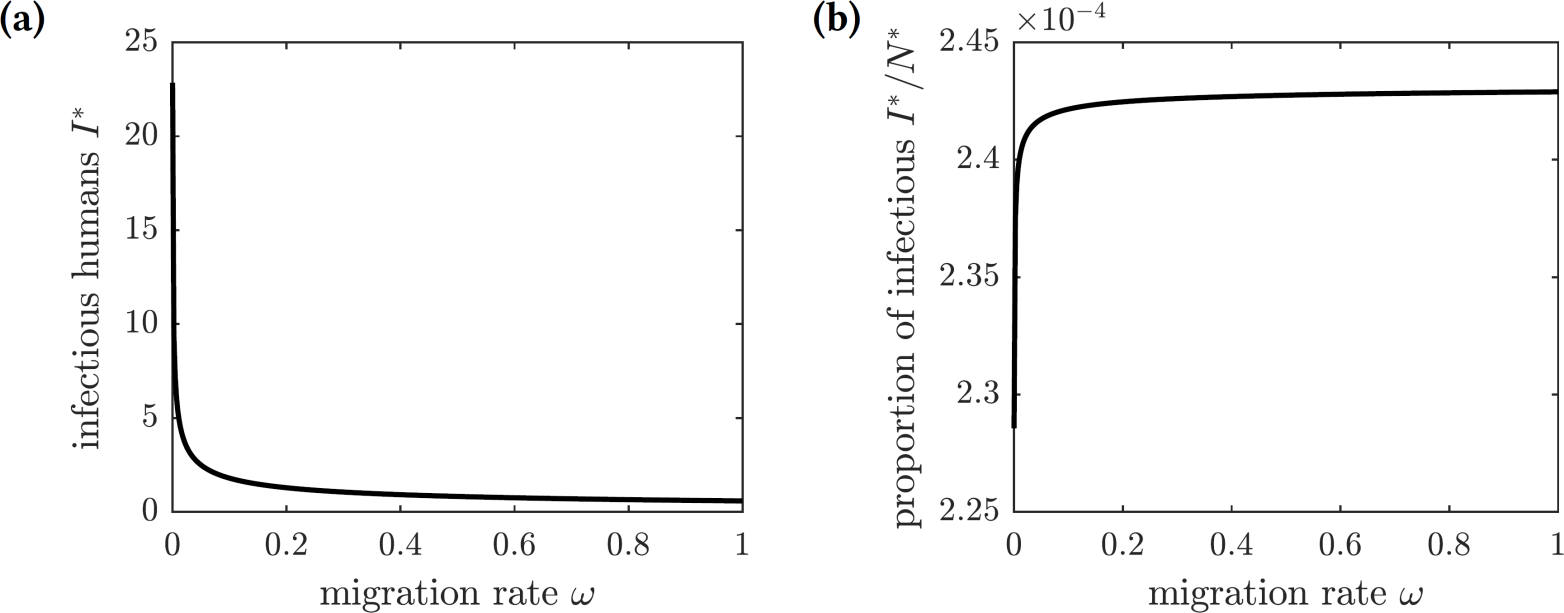
The graphs of the (A) number and (B) proportion of symptomatically infectious individuals in the population as functions of the emigration rate *ω*. Increased migration levels result in fewer infectious individuals overall but a greater proportion of infectious individuals in the population.

Figure 6 shows the graphs of the number and proportion of symptomatically infectious individuals in the population as functions of the emigration rate *ω*. As more individuals leave the island, the overall population level declines, and hence there are fewer infected individuals. However, the infection spreads faster among the remaining inhabitants, resulting in a greater proportion of infected individuals in the population. The proportion of symptomatically infectious individuals grows with the migration rate, but it asymptotically approaches the value

(27) \begin{eqnarray*}\lim _{\omega \rightarrow \infty } \frac{{I}^{\ast }}{{N}^{\ast }} = \frac{\phi {\mu }_{1}{\lambda }_{1}}{(\gamma +{\mu }_{1})({\lambda }_{1}+{\mu }_{1})} .\end{eqnarray*}

We next consider a game-theoretic model of individual migration decisions. Suppose that the population adopted the emigration rate *ω*_pop_. A focal susceptible individual is presented with a choice to either migrate or not migrate. Each of the two strategic choices carries a corresponding payoff: *E*_m_ for migrate and *E*_nm_ for not migrate, given by

(28) \begin{eqnarray*}{E}_{\text{m}}({\omega }_{\text{pop}}) & =-{C}_{b}-{\omega }_{\text{pop}}{C}_{s},\text{and} & {E}_{\text{nm}}({\omega }_{\text{pop}}) & =-\pi ({\omega }_{\text{pop}}){C}_{i},\end{eqnarray*}

where *C*_*b*_ is the base (fixed) cost of migration, *C*_*s*_ is the scaling cost of migration, *C*_*i*_ is the cost of the (symptomatic) chikungunya infection, and *π*(*ω*_pop_) is the probability of getting infected given the population emigration rate *ω*_pop_. We assume that the cost of emigration is an increasing function of the migration rate because of the limited immigration potential of Mauritius: the more individuals migrate to Mauritius, the harder it becomes to find housing and jobs. For simplicity, we model the increasing emigration cost as a linear function of the migration rate. The probability of getting infected and incurring the cost of a symptomatic chikungunya infection if remaining on Reunion Island is the transition probability from the susceptible class *S* to the symptomatically infectious class *I*:

(29) \begin{eqnarray*}\pi ({\omega }_{\text{pop}})= \frac{{f}_{1}({\omega }_{\text{pop}})}{{\mu }_{1}+{f}_{1}({\omega }_{\text{pop}})} \frac{\phi {\lambda }_{1}}{{\mu }_{1}+{\lambda }_{1}} .\end{eqnarray*}

**Figure 7:**
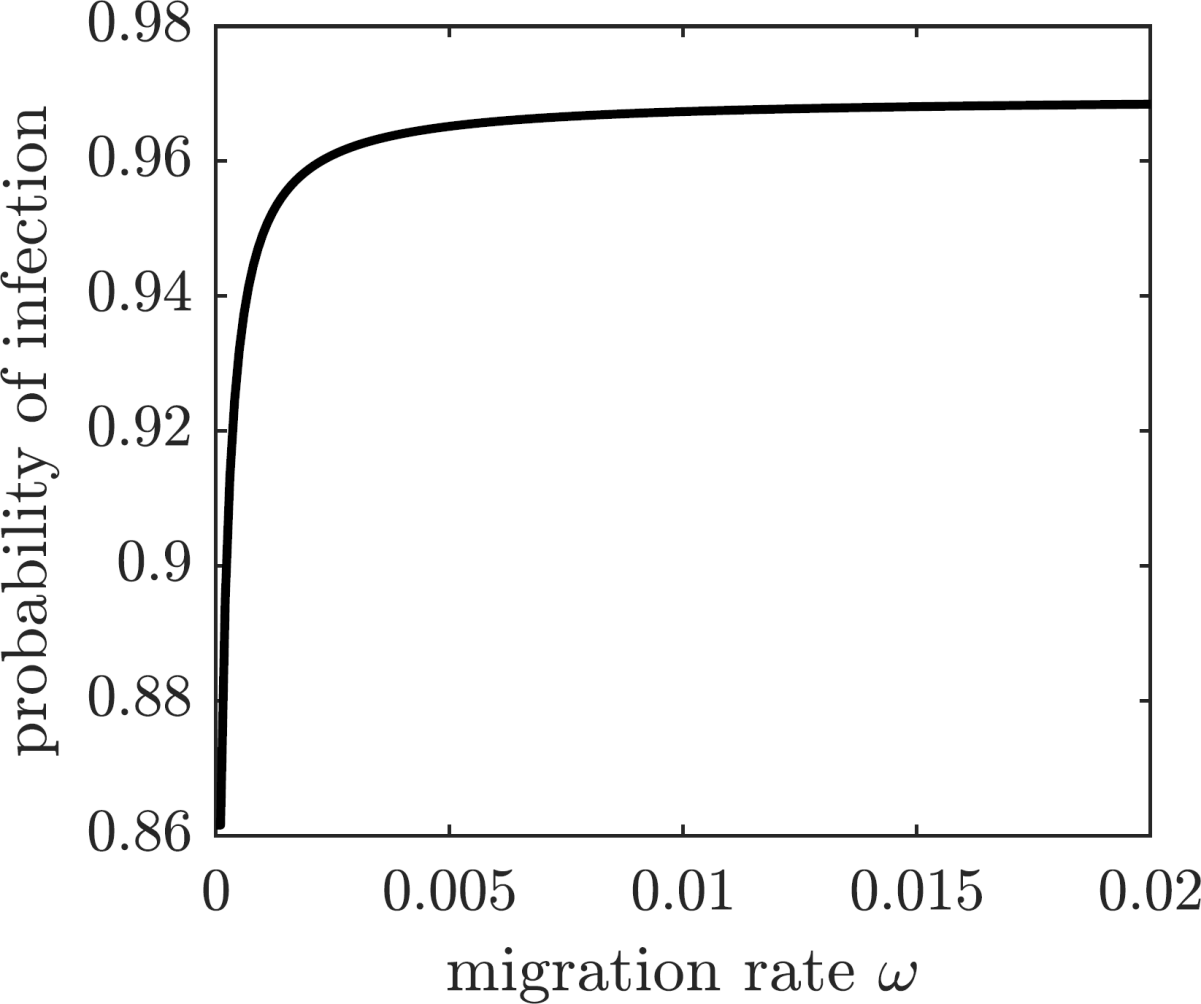
The probability of symptomatic chikungunya infection for a susceptible individual on Reunion Island is increasing with the emigration rate *ω*.

This probability is an increasing function of the emigration rate because each of the remaining susceptible individuals faces a higher risk of infection (cf. Fig. 5); the graph is shown in Fig. 7.

To find conditions when a focal susceptible individual should emigrate to Mauritius or remain on Reunion, we scale the payoffs in Eq. (28) by 1∕*C*_*i*_ and obtain

**Figure 8:**
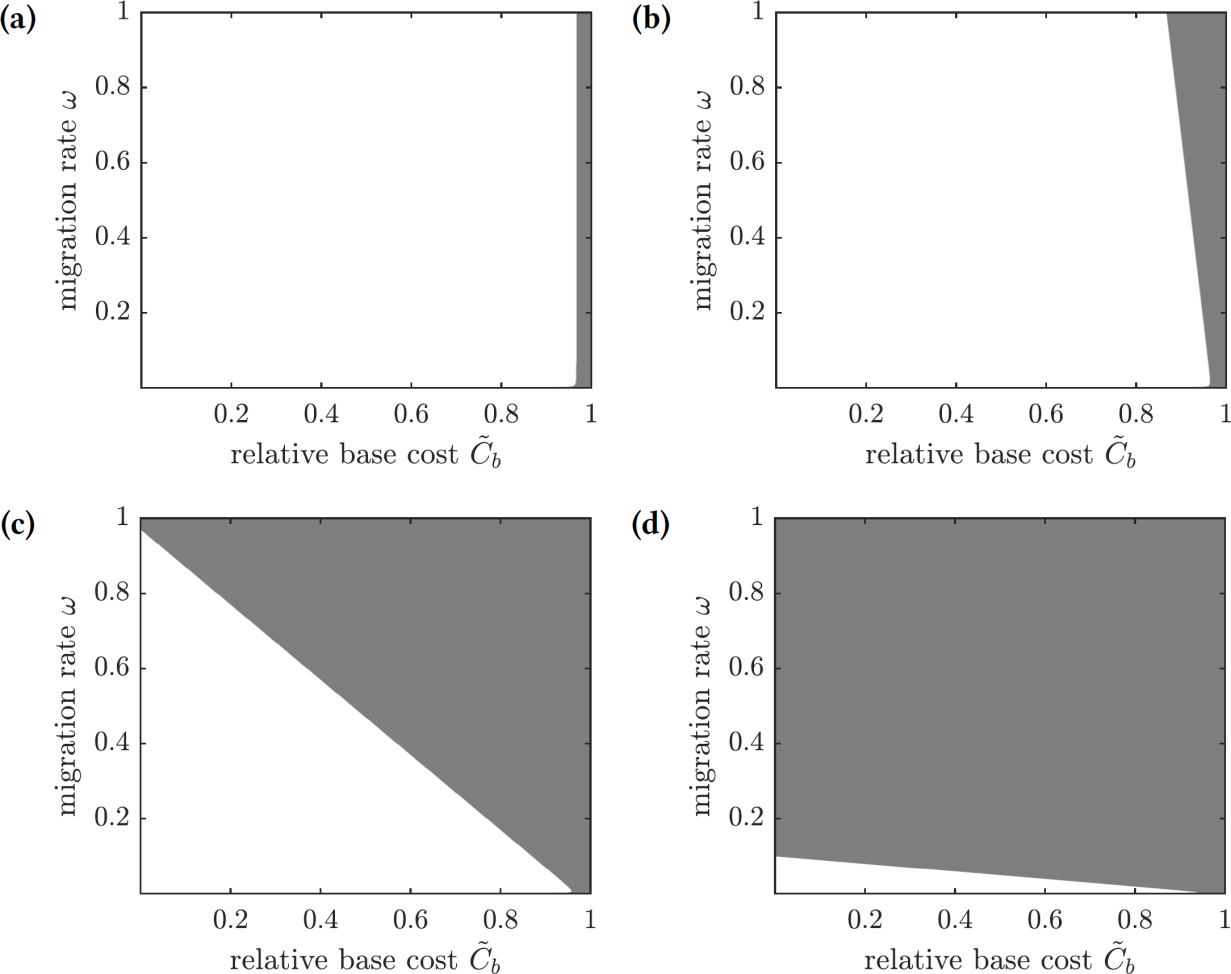
The regions in the $({\tilde {C}}_{b},\omega )$-parameter space showing whether a focal susceptible individual should emigrate to Mauritius or remain on Reunion. Color code: white—emigrate, gray—stay. (A) ${\tilde {C}}_{s}=0$, (B) ${\tilde {C}}_{s}=0.1$, (C) ${\tilde {C}}_{s}=1$, (D) ${\tilde {C}}_{s}=10$.

(30) \begin{eqnarray*}{E}_{\text{m}}=-\tilde {{C}_{b}}-{\omega }_{\text{pop}}\tilde {{C}_{s}},\text{and}{E}_{\text{nm}}=-\pi ({\omega }_{\text{pop}}),\end{eqnarray*}

where ${\tilde {C}}_{b}$ and ${\tilde {C}}_{s}$ are relative base and scaling costs of emigration, respectively. A susceptible individual should emigrate when the relative cost of doing so is less than the probability of getting infected: $\tilde {{C}_{b}}+{\omega }_{\text{pop}}\tilde {{C}_{s}}\lt \pi ({\omega }_{\text{pop}})$, and the individual should remain on the island otherwise. The regions in the $({\tilde {C}}_{b},\omega )$-parameter space corresponding to the best choice for a focal individual for several values of ${\tilde {C}}_{s}$ are shown in Fig. 8. If the scaling cost of emigration *C*_*s*_ is negligible (i.e., the cost of emigration does not depend on the number of emigrating individuals), then the best strategy of a susceptible individual is to emigrate as long as the relative base cost of emigration $\tilde {{C}_{b}}$ is sufficiently small (Fig. 8A). On the other hand, as the relative scaling cost of emigration $\tilde {{C}_{s}}$ grows, the individual’s decision to emigrate starts to depend on the emigration decisions of other individuals (Figs. 8B–8C), until it becomes unprofitable to emigrate regardless of the relative base cost of emigration if the emigration rate is too high (Fig. 8D).

### Optimal levels of mandatory emigration

Finally, we consider the potential impacts on the chikungunya epidemic on Reunion Island of coordinated emigration efforts. A mandating organization attempts to minimize overall costs, which are comprised of the cost of treatment of symptomatically infected individuals and the relocation costs of emigrating individuals. To estimate the number of emigrating susceptible individuals, we consider the difference between the total population size at equilibrium without emigration (*N*^∗^ = Λ_1_∕*μ*_1_) and the total population size at equilibrium given the population migration rate *ω* (this expression is given in the first equation of (22)); we denote this difference by ${N}_{\omega }^{\ast }$.

The payoff of the emigration policy with migration rate *ω* is given by

**Figure 9:**
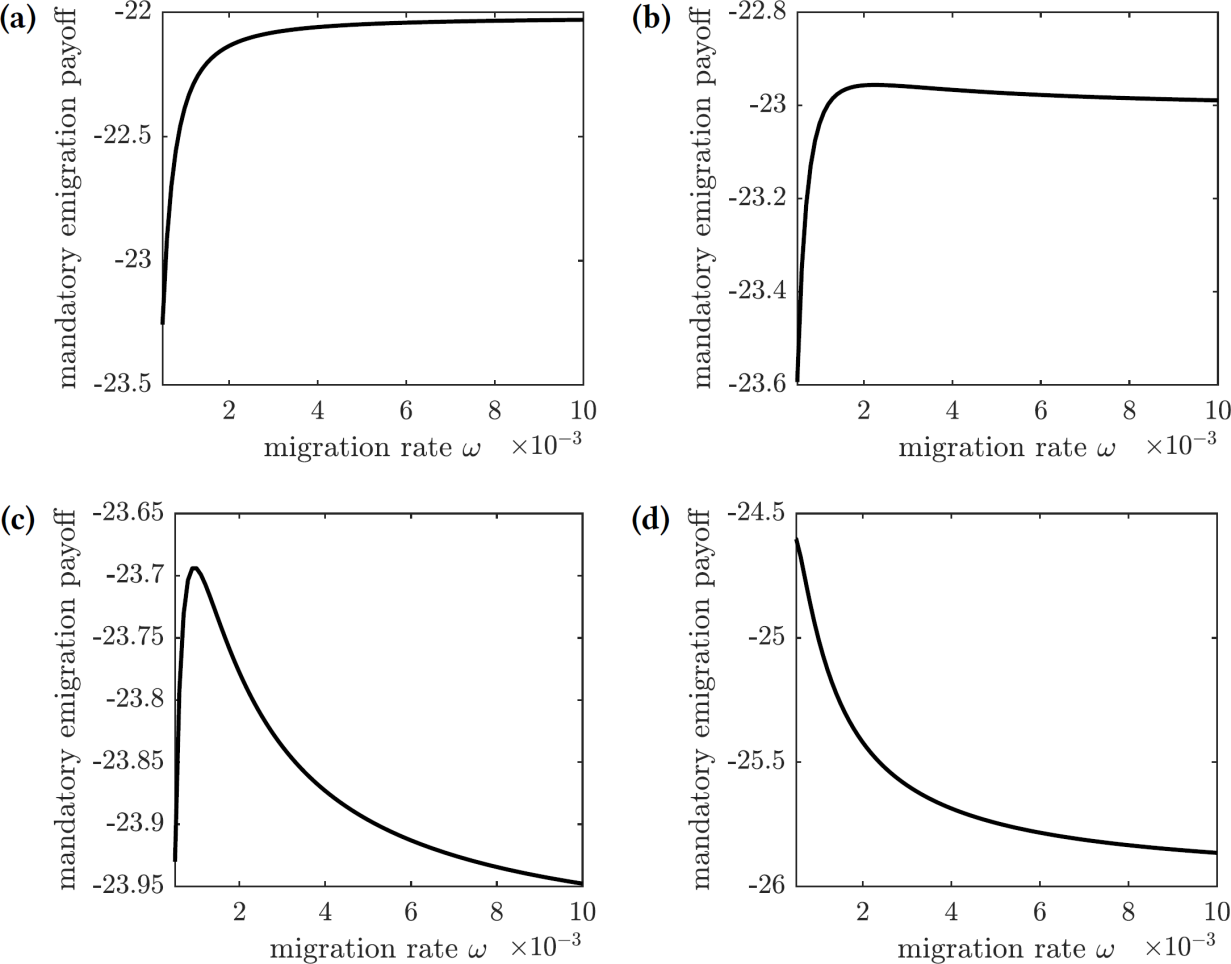
The overall cost of the mandated emigration policy as a function of the migration rate *ω*. (A) ${\tilde {C}}_{m}=0.00022$, (B) ${\tilde {C}}_{m}=0.00023$, (C) ${\tilde {C}}_{m}=0.00024$, **(d)** ${\tilde {C}}_{m}=0.00026$.

(31) \begin{eqnarray*}E(\omega )=-{I}^{\ast }-{\tilde {C}}_{m}{N}_{\omega }^{\ast },\end{eqnarray*}

where ${\tilde {C}}_{m}={C}_{m}/{C}_{i}$ is the cost of migration relative to the cost of infection. The graphs of this function for several values of ${\tilde {C}}_{m}$ are shown in Fig. 9. There are three qualitatively different outcomes:

1. For very low relative migration cost (${\tilde {C}}_{m}\leq 0.00022$), higher migration rates result in smallest overall costs; however, the near-optimal costs are quickly achieved by small values of migration rate (*ω* = 0.01)—see Fig. 9A.
2. There is a small interval of the relative migration cost values ($0.00023\leq {\tilde {C}}_{m}\leq 0.00025$) where the optimal cost is achieved in the interior for very small values of the migration rate (*ω* < 0.002)—see Figs. 9B and 9C.
3. For all sufficiently large values of the relative migration cost (${\tilde {C}}_{m}\geq 0.00026$), it is best not to allow individuals to emigrate from the island—see Fig. 9D.

In practice, however, the cost of emigration (such as relocation from Reunion to Mauritius) is usually comparable to or higher than the cost of the symptomatic chikungunya infection. Therefore, the scenario shown in Fig. 9D is the most realistic one: it is best not to allow susceptible individuals to leave the island during the outbreak.

## Discussion

We investigated potential implications of both voluntary and mandatory intervention measures to fight the chikungunya outbreak on Reunion Island. Susceptible individuals may either prevent the infection by using insect repellent and hence reduce the frequency of mosquito bites, or leave Reunion Island and emigrate to neighboring Mauritius. We adopted a version of a previous epidemiological model of the chikungunya transmission on Reunion Island by Yakob & Clements (2013). The epidemiological model informed the payoff functions in the game-theoretic models of individual and centralized decisions on the level of adoption of the protective measures. We found that the two protocols resulted in qualitatively different predictions concerning optimal allocations, with the latter measure creating an additional hazard for non-participants.

Voluntary participation in the two intervention measures produced opposite population-level effects. The more susceptible individuals spray themselves with insect repellent, the less likely the infectious mosquitoes generate new human infections before they die. Consequently, higher adoption levels of insect repellent usage in the population resulted in lower basic reproduction number values for the disease. Individuals using repellent provide (near) herd-immunity-effect benefits to the entire population. In contrast, if susceptible individuals vacated the island, then susceptible mosquitoes were more likely to bite infectious humans as a percentage of the remaining population, thus increasing the disease prevalence among mosquitoes. The remaining susceptible individuals subsequently faced an increased risk of contracting the infection from a mosquito bite. Increased migration levels resulted in drastically elevated basic reproduction number values. Thus, the impact of voluntary emigration is similar to the tragedy-of-the-commons effect: while being potentially beneficial to specific individuals, it hurts the remaining islanders.

The mandated repellent usage protocol resulted in the same outcome as the voluntary (i.e., selfishly rational) compliance scenario if the cost of the preventive measure relative to the cost of the disease was too high: it was best to bypass the repellent usage altogether. But if the relative cost of protection was sufficiently low, so that repellent usage was warranted, then the two scenarios effected different outcomes. In the voluntary compliance case, the population repellent usage fell short—albeit not by much—of the herd immunity threshold. In the mandated protocol case, reaching the herd immunity usage level and thus eradicating the disease was most effective.

That voluntary adoption of preventative measures against an infectious disease falls short of the herd immunity threshold has also been observed in other studies (Geoffard & Philipson, 1997; Bauch & Earn, 2004; Dorsett et al., 2016). Yet looking at a mandated repellent usage scenario revealed that a mandatory protocol might have eliminated the epidemic if the relative cost of the preventive measure was sufficiently low.

Mandatory emigration from Reunion Island demonstrated that this preventive measure made sense for the public benefit only when the cost of relocation was significantly lower than the public cost of infection. Since this mathematical assumption is not likely to hold in practice, the model predicted that it was best to avoid migration of susceptible individuals from the island. The potentially high cost of relocating susceptible individuals away from the epidemic was not compensated by the minimal decrease in the number of infected individuals.

The qualitative differences in optimal behavior under the two alternative treatment protocols invite further examination of our model’s behavior and assumptions. Both evacuation/emigration of the human populace and the use of repellent reduce the pool of potential blood hosts for the mosquito population; however, they produce contrasting effects on the force of infection. A base assumption in the model is that each insect has a consistent average number of encounters with humans over a given time span. Repellent usage directly decreases the force of infection by deterring biting upon encounter—it is this feature of “wasted” encounters that permits the development of herd immunity. In contrast, reduction in the size of the standing human population elevates the force of infection by increasing the number of encounters an individual human experiences. Secondarily, this results in increased prevalence of the disease in the vector-population as their blood hosts are more likely to be infected. We hypothesize that distinct protocol results depend upon the presence of (1) a distinct vector population; (2) an assumption of constant predation encounters for vectors; (3) the proportional allocation of encounters across humans; (4) an inability of vectors to pre-judge encounters and thereby shift towards more palatable hosts; and (5) a secondary food source to support constant recruitment of new vectors. An experimental study may be warranted to verify the validity of our assumptions. We also propose a followup study to this paper that focuses specifically on the dynamic analysis of the force of infection as these assumptions are introduced or removed.

## Conclusions

There are several additional directions in which our model can be improved. First, we are assuming that individuals possess complete information regarding the prevalence of the disease and the costs of protection relative to infection. But individuals rarely have access to the exact disease prevalence data, and hence they may only guess the relevant numbers. Second, the cost of intervention (such as using insect repellent) and the cost of the disease must be estimated individually. These costs include both direct costs such as paying for repellent or medical treatment, and indirect costs such as potentially harmful side effects of the chemicals in repellent or morbidity risks of the infection. Additionally, different individuals may have various opinions about the risk of using repellent or getting infected with chikungunya virus. Building these uncertainties into the model should allow a broader outlook at different strategies to combat such outbreaks.

Moreover, our model assumes that the population has reached an equilibrium with respect to the disease dynamics. But reaching this equilibrium usually occurs on a different timescale compared to individual preventive actions. For example, individuals could be more likely to participate in preventive efforts when the epidemic is at its peak rather then when the disease reached the endemic state. A dynamic model where susceptible individuals inform their preventive decisions on the current state of the prevalence of the disease which, in turn, affects the dynamics of the disease transmission, should present a more realistic analysis of selfish individual decisions to prevent the infection.

##  Supplemental Information

10.7717/peerj.10151/supp-1 Supplemental Information 1 Matlab code for computing and visualizing the effects of voluntary emigration from the island

10.7717/peerj.10151/supp-2 Supplemental Information 2 Matlab code for computing and visualizing the outcomes of the game-theoretic model of optimal repellent usage

10.7717/peerj.10151/supp-3 Supplemental Information 3 Matlab code for computing and visualizing the outcomes of the game-theoretic model of voluntary emigration

## Additional Information and Declarations

### Data Availability

The following information was supplied regarding data availability: All MATLAB code that was used to obtain the results and create figures is available in the Supplemental Files.
